# On the origin of pure optical rotation in twisted-cross metamaterials

**DOI:** 10.1038/srep30307

**Published:** 2016-07-26

**Authors:** Lauren E. Barr, Ana Díaz-Rubio, Ben Tremain, Jorge Carbonell, José Sánchez-Dehesa, Euan Hendry, Alastair P. Hibbins

**Affiliations:** 1 Electromagnetic Materials Group, School of Physics, University of Exeter, Stocker Road, Exeter, EX4 4QL, United Kingdom; 2 Wave Phenomena Group, Department of Electronics Engineering, Universitat Politècnica de Valéncia, Camino de Vera S/N. (Edificio 7F1), ES-46022 Valencia, Spain

## Abstract

We present an experimental and computational study of the response of twisted-cross metamaterials that provide near dispersionless optical rotation across a broad band of frequencies from 19 GHz to 37 GHz. We compare two distinct geometries: firstly, a bilayer structure comprised of arrays of metallic crosses where the crosses in the second layer are twisted about the layer normal; and secondly where the second layer is replaced by the complementary to the original, i.e. an array of cross-shaped holes. Through numerical modelling we determine the origin of rotatory effects in these two structures. In both, pure optical rotation occurs in a frequency band between two transmission minima, where alignment of electric and magnetic dipole moments occurs. In the cross/cross metamaterial, the transmission minima occur at the symmetric and antisymmetric resonances of the coupled crosses. By contrast, in the cross/complementary-cross structure the transmission minima are associated with the dipole and quadrupole modes of the cross, the frequencies of which appear intrinsic to the cross layer alone. Hence the bandwidth of optical rotation is found to be relatively independent of layer separation.

Optical rotation, *Φ*, is the rotation of linearly polarized radiation associated with intrinsic chirality of objects^1^, and is a phenomenon with important applications in analytical chemistry, biology, and crystallography^2^. Electromagnetic interactions in chiral media can be described by an infinite series of multipolar terms, but in the simple dipole approximation optical rotation arises when electric and magnetic dipole moments of a resonator are parallel and out of phase^3^, described by


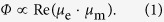


For optically active molecules, this coupling between the electric (*μ*_e_) and magnetic (*μ*_m_) dipoles is usually very weak, due to a mismatch between the length scales of the molecules and the wavelength of light^3^.

Recently, new possibilities have emerged using so-called “chiral metamaterials” (CMs). The most common embodiment of CMs is a regular array of subwavelength elements (“meta-atoms”), but pairs or groups of achiral elements arranged in a handed configuration to make a “metamolecule” which is chiral have also been considered^4^. Due to their interdependent electric and magnetic responses, these materials can exhibit different refractive indices for right and left circularly polarised radiation, leading to a splitting of transverse modes (which are otherwise degenerate for appropriate geometries) and a region of negative refraction for one handedness of radiation. Since this discovery, CMs have attracted a lot of attention as an alternative to the traditional combination of split-ring and dipole resonators for negative refractive index materials^5^ and can be used for the design of perfect lenses^6^. Whereas a single layer of 3D chiral elements will intrinsically demonstrate circular dichroism, 2D elements need to be stacked to produce a bulk metamaterial with a similar response. Circular dichroism and optical rotation can be optimised by modifying the geometry of the meta-atoms forming each layer of the CM, and are typically orders of magnitude stronger than for molecular chiral materials^2^. Several layered structures exhibiting large optical rotation have been proposed that have differing designs: e.g. rosettes^2^, gammadions^7^ or U-shaped split resonators^4^. One of the simplest cases is a bilayer system with twisted crosses, which shows strong optical rotation and negative refractive index^8^,^9^,^10^,^11^. However, the optical activity of all of these designs relies on evanescent coupling between the meta-atoms to yield metamolecules, which typically leads to a highly dispersive optical behaviour^12^,^13^.

Despite this, dispersionless optical rotation over a broad frequency range between resonances has recently been demonstrated in studies of bilayer structures formed from arrays with twisted crosses coupled to their complementary cross (c-cross), as well as chiral geometries based on 3D metallic helices^14^. The cross/c-cross structure has the added advantage that optical rotation occurs over a frequency range which also exhibits high transmission. Hannam *et al*. demonstrated the optical rotation of a cross and its c-cross embedded in a circular waveguide^12^, while Zhu *et al*. computationally modelled arrays of twisted crosses and c-crosses^13^. While all of these studies found large optical rotations (note that Zhu *et al*. reported an optical rotation ~164°, which is equivalent to a rotation of polarisation ~16°), the most striking feature is the broadband nature of the effect. However, a thorough explanation of this phenomenon is currently missing from the literature.

In this letter, we report a combined experimental and computational study of optical rotation in different types of twisted cross metamaterials in free space in order to better understand the phenomenon. We compare two distinct geometries: firstly, a bilayer structure comprising of square arrays of metallic crosses separated by a thin dielectric sheet, where the crosses in the second layer are twisted with respect to the layer normal, and a second system where the lower layer is instead an array of twisted c-crosses. We show that the optical rotation in the cross/cross metamaterials can be understood by considering coupled electric dipole resonances between the two layers that gives rise to an operational bandwidth which is very strongly dependent on layer separation. In the cross/c-cross structure, meanwhile, we show that optical rotation occurs in a region between the dipolar mode and a higher order resonance of the cross element. Since modes defining the periphery of the region of optical rotation in the cross/complementary-cross structure appear intrinsic to the cross layer alone, the bandwidth of optical rotation is found to be relatively independent of layer separation. This gives rise to a broad region of pure, near dispersionless optical rotation, spanning a range from 19.0 GHz to 37.7 GHz, and coinciding with a peak in transmission.

## Experimental Measurement of Optical Rotation

**Figure 1:**
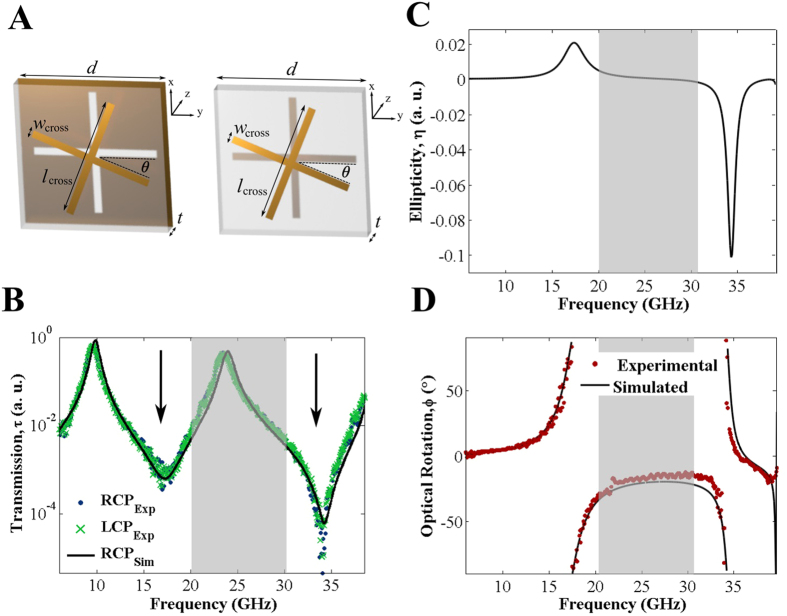
Comparison of experimental and simulated results from cross/complementary-cross array. (**A**) Diagrams of a unit cell of the cross/cross (left) and cross/complementary-cross (right) bilayer under investigation; in both cases the dimensions are *d* = 7.5 mm, *t* = 406 μm, l_cross_ = 6.60 ± 0.05 mm, *w*_cross_ = 0.375 ± 0.05 mm, *θ* = 22.5°; (**B**) Experimental and simulated transmission amplitude of right- and left-handed circularly polarised waves through a cross/complementary-cross bilayer with etching inconsistencies included in the model; only the simulated RCP is plotted as the transmission for LCP overlays it except at the resonant transmission minima, indicated by black arrows, at which optical rotation occurs; shaded region shows frequency range of pure optical rotation; **(C**) Prediction of ellipticity from FEM model with losses included; (**D**) Experimental and simulated optical rotation.

The schematic of a metamolecule of the cross/c-cross chiral bilayer under investigation is shown in Fig. 1(A), and is similar to that reported in ref. 15. The metamaterial consists of a double-sided, copper-clad dielectric sheet etched to produce a square array of copper crosses on one side, and an array of cross-shaped holes on the other. The elements in the latter have almost identical dimensions, are aligned with and arranged on the same lattice as the first, but the individual elements are each rotated in the plane about their centre. Etching inconsistencies during fabrication resulted in a discrepancy between the dimensions of the cross and c-cross elements of around 0.05 mm, which has been taken into account in the FEM model (HFSS^16^). This results in a small shift of resonant frequencies compared to the ideal case described in section 2.B. The dimensions of the cross/cross bilayer sample discussed later are identical, and the effects of over-etching are neglected. The thickness of the dielectric is 0.406 mm with permittivity, *ε_d_* = 3.02(1 + 0.02*i*). The lattice spacing is *d* = 7.5 mm, the length and the width of the crosses are l_cross_ = 6.60 ± 0.05 mm and *w*_cross_ = 0.375 ± 0.05 mm, and the element rotation angle is *θ* = 22.5°. The sample is formed by 50 unit cells in the *x*-direction and 50 in the *y*-direction, although in the finite element method (FEM) model the array is assumed to be infinite in both directions.

We obtain the circularly polarised transmission coefficients by measuring the four linear, complex transmission coefficients (*t*_xx_, *t*_xy_, *t*_yy_, and *t*_yx_). In the experimental setup, linearly polarized microwave radiation impinges at normal incidence upon the sample from a rectangular waveguide horn antenna, and the transmitted beam is collected by a second rectangular horn antenna. Both antennas are connected to a vector network analyser and can be azimuthally rotated by 90° to permit the polarisation-dependent measurements required. The simulations are performed using a with one unit cell and periodic conditions in the *x*- and *y*- directions. Floquet ports are employed in the model, and the complex transmission coefficients are obtained from these ports. The circular transmission amplitude coefficients, *τ*, for each polarization can be calculated from the complex linear transmission coefficients^11^ using


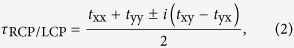


where the subscript on *τ* denotes left (LCP) or right (RCP) circular polarizations, which correspond to the + or − sign in the expression respectively. The optical rotation, *φ*, and the ellipticity, *η*, are defined respectively as


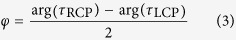


and, assuming there is no polarisation conversion,


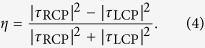


In Fig. 1(B) the experimental amplitudes of the left and right circular polarization transmission coefficients (circles and crosses) are compared with numerical simulation (lines). Good agreement between theory and experiment is observed, with a maximum transmission intensity of 0.86 at 9.8 GHz. A second maximum occurs at 24.0 GHz, bounded by two clear transmission minima at 17.2 GHz and 34.2 GHz. Figure 1(C) shows the predictions of the ellipticity of transmitted radiation from the FEM model. We see the maxima in ellipticity arise from the small difference between the left and right circularly polarised transmission intensities near the transmission minima (arrows in Fig. 1(B)). Note that we do not plot experimental results for ellipticity, as the signal to noise ratio is poor when measuring near zero transmission close to resonance. An important point to note is that when losses are removed from the permittivity of the dielectric layer the peaks in ellipticity disappear, as ellipticity arises from a difference in absorption for RH and LH radiation which vanishes when losses are removed.

The optical rotation, obtained via the phase measurements using Eq. 3 is shown in Fig. 1(D). In the frequency region in-between the transmission minima we observe a broad, near dispersionless optical rotation, marked as a shaded region in Fig. 1(B–D). This is a key result which will be discussed in the remainder of the paper. As discussed in refs 11,15, broadband optical rotation accompanied by near-zero ellipticity is rather unusual. Moreover, the region of dispersionless optical rotation (~19°) is bounded by the transmission minima associated with two resonances of the system (marked by arrows in Fig. 1(B)). It is the nature of these resonances, and how they define the behaviour of the structure, that we wish to elucidate in the discussion below.

## Numerical Modelling and Discussion

### Cross/Cross Bilayer

**Figure 2:**
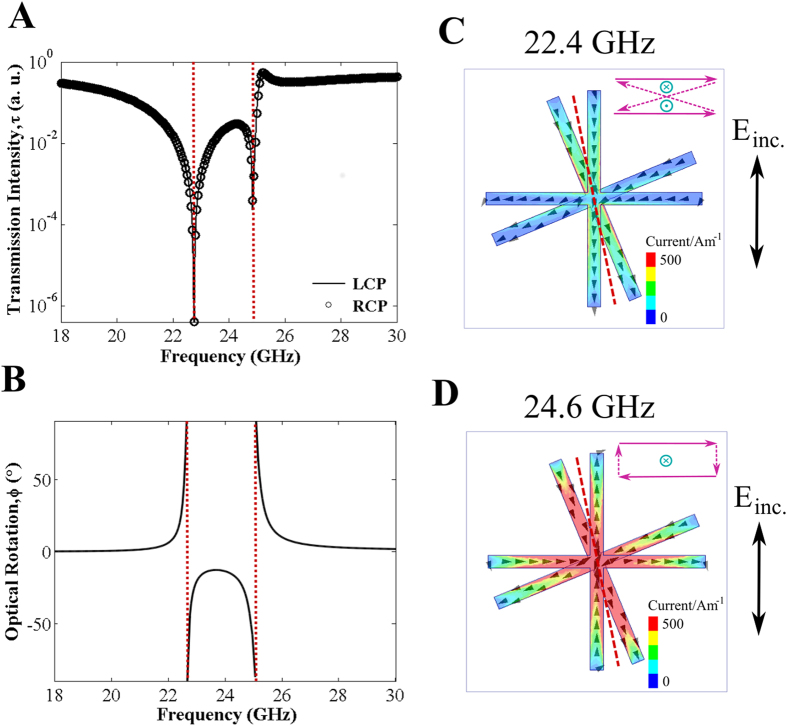
Simulated demonstration of pure optical rotation on a cross/cross array. (**A**) Modelled transmission amplitude of left- and right-handed circularly polarised waves with etching inconsistencies neglected; (**B**) Optical rotation of transmitted radiation; dotted red lines indicate resonant modes; Direction of current (arrows) and surface current density (colour) in the upper and lower elements of one unit cell at (**C**) *f* = 22.5 GHz and (**D**) *f* = 24.6 GHz, corresponding to resonant dips in transmission; inset in each, purple arrows represent electric dipoles and blue arrows, magnetic dipoles in a cross-section through the dielectric in the mid-rotation plane, marked by the dashed red line.

In order to determine the underlying mechanism behind the broadband optical rotation, we consider first optical rotation arising from a more conventional twisted cross/cross array. Figure 2(A) shows the transmitted intensities of left- and right-handed circularly polarised waves (LCP and RCP) through a metamaterial comprised of left-handed metamolecules (defined as having an anticlockwise rotation from top to bottom crosses), calculated using FEM. We observe two resonant modes at 22.5 GHz and 24.6 GHz. These resonant frequencies are defined by transmission minima, as expected for an array of unconnected metallic antennas^17^. We can characterize the nature of these modes by considering the currents flowing in the arms of the crosses at these frequencies. We observe strong currents in the cross arms aligned parallel to the incident electric field, as shown in Fig. 2(C,D). For the two different resonance frequencies we observe currents with a single maximum at the mid-length of the arms. This is characteristic of an electric dipole resonance of a cross. For the lower frequency mode, the current direction at any point in the phase cycle is the same in the upper and lower cross (i.e. the currents are in phase.), while for the upper frequency mode, the currents in the two layers point in opposite directions (i.e. they are out of phase). We therefore label these as the symmetric (22.5 GHz) and antisymmetric (24.6 GHz) resonances of the coupled cross system.

Chiral interactions such as circular dichroism and optical rotation arise due to multipolar interactions normally dominated by alignment of magnetic and electric dipole moments (Eq. 1)^4^. From the classical viewpoint this means that the electric field of the incident radiation can induce a magnetic dipole moment in the structure parallel to the incident electric field and that the magnetic field of the incident radiation can induce a corresponding electric moment parallel to the incident magnetic field. While the materials that form our cross meta-atoms are themselves nonmagnetic, an effective magnetic dipole can be generated by the electric dipole coupling giving rise to a current loop (completed by displacement currents in the dielectric) and in-plane magnetic dipole moments. We represent these current loops in the insets of Fig. 2(C,D), where the inset shows a projection of electric and magnetic dipoles onto the mid-rotation plane (dashed red line).

For the higher frequency, antisymmetric dipole resonance, the origin of the magnetic dipole moment is relatively easy to understand. Following the interpretation of Kenanakis *et al*.^18^, the displacement fields link the ends of the upper cross arm with the ends of the lower cross arm, forming current loops. This gives rise to a single magnetic dipole moment with a direction lying in the plane of the sample. In un-rotated cross/cross structures (which have mirror symmetry) this magnetic dipole lies perpendicular to the electric dipoles of the crosses themselves, and, according to Eq. 1, we will not observe optical rotation due to this mode, as expected from the symmetry. However, when one cross is twisted with respect to the other, partial alignment of the electric and magnetic dipoles occurs, leading to the onset of optical rotation. For the symmetric (lower frequency) mode, one might expect no optical rotation, since the currents are parallel in the arms of the two crosses. However, in Fig. 2(A) we clearly also observe an optical rotation resonance associated with this mode. Here, displacement currents between upper and lower crosses create a figure-of-8 current loop (see inset of Fig. 2(C)), which can be thought of as two counter-rotating loops, each with an associated magnetic moment. Without a twist between upper and lower crosses these magnetic moments are antiparallel and hence cancel. However, a rotation of one cross with respect to the other results in a net magnetic moment which is parallel to the net electric dipole, again satisfying Eq. 1, and resulting in optical rotation.

In each case, the sign of optical rotation is determined by the sign of the phase difference between electric and magnetic moments, i.e., if the electric moment leads the magnetic moment the optical rotation will be positive or right-handed in nature, while if the magnetic moment leads the optical rotation will be negative and described as left-handed. On approaching the symmetric, lower frequency resonance, the optical rotation we observe is positive. At the resonance, the transmission drops to almost zero and the currents in the top and bottom arms of the cross undergo a sharp 180° phase change; the handedness of the optical rotation reverses, as now the magnetic moment is leading the electric moment by 90°. However, it is interesting to note that, as seen in Fig. 2A, the sense of optical rotation is reversed at the upper and lower frequency modes, meaning that the direction of polarization rotation is reversed. This gives rise to a frequency band in-between the two resonances where the optical rotation does not change sign. This observation provides a key insight into the conditions necessary in such a system for pure optical rotation (i.e., without ellipticity) to be observed. In the following section we discuss how some of the reasoning outlined above can be applied to the complementary case of a cross/c-cross structure.

### Cross/Complementary-Cross Bilayer

Replacing the lower cross layer with its complement (cross shaped holes in a continuous metal sheet) has been shown to result in similar dispersionless optical activity^12^. While one might expect the underlying mechanism behind this optical rotation to be the same as that for the cross/cross structure, we find that the pair of resonances involved are of a fundamentally different nature.

**Figure 3:**
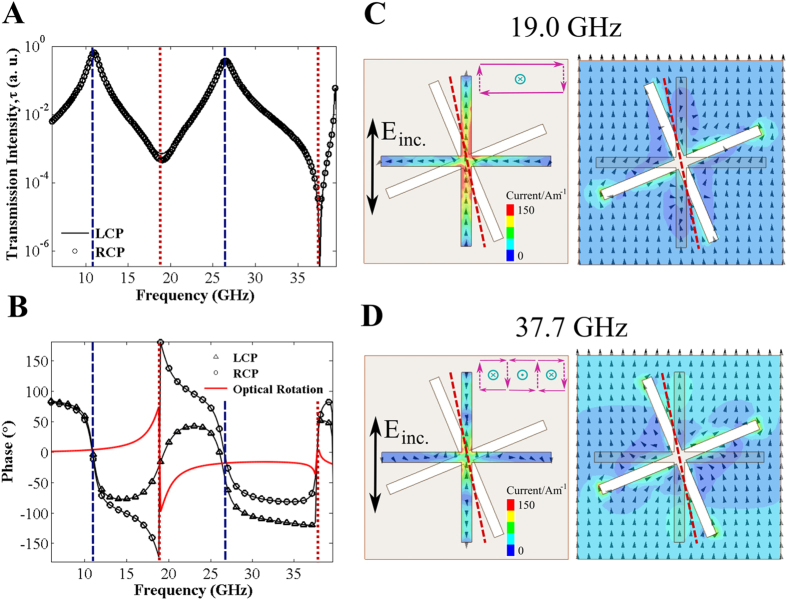
Simulated demonstration of wideband pure optical rotation on a cross/complementary-cross array. (**A**) Modelled transmission of left- and right-handed circularly polarised waves with etching inconsistencies neglected; (**B**) Phase on transmission of left- and right-handed circularly polarised radiation (marked with triangles and circles respectively) and the resulting optical rotation (solid red line); dashed blue lines indicate resonant maxima and dotted red lines, resonant minima; Direction of current (arrows) and surface current density (colour) in the upper (left) and lower (right) elements of one unit cell at (**C**) *f* = 19.0 GHz and (**D**) *f* = 37.7 GHz, corresponding to peaks in transmission; black arrow shows the polarisation of incident electric field; inset in each, purple arrows represent electric dipoles and blue arrows, magnetic dipoles in a cross-section through the dielectric in the mid-rotation plane, marked by the dashed red line.

In Fig. 3(A) we plot the transmission spectra for the cross/c-cross structure. In addition to transmission minima associated with resonances of the crosses, marked by red dotted lines, we now also have transmission maxima at the frequencies marked by the blue dashed lines. For ease of interpretation, we have neglected the discrepancy in upper and lower element size described in section 1 in this model. Transmission maxima are a characteristic feature of arrays of apertures in a conducting sheet^19^, and are therefore primarily associated with resonances of the complementary cross layer. Figure 3(B) shows the transmitted phase of the double layer system. Similarly to the cross/cross system, the band of dispersionless optical rotation in Fig. 3(B) is bounded by two resonance minima, each rotating the polarisation plane in opposite directions. It should be noted that discontinuities in phase near transmission minima is a phenomenon widely observed in optics and plasmonics^20^. However, phase discontinuities at transmission minima play a particularly important role in chiral media: Gorkunov *et al*. recently showed that transmission minima in chiral metamaterials can give rise to very strong circular dichroic and optical rotatory effects, which must be accounted for in Kramers-Kronig relations by including so-called Blaschke terms^21^. Figure 3(C) shows that the lower frequency resonance minimum is associated with the dipolar excitation of the cross. However, because we have removed the second cross from the unit cell, replacing it with the complementary layer, one does not expect a higher frequency antisymmetric dipole resonance, as was the case for the cross/cross above. Instead, we see the second minimum in transmission is associated with a higher order excitation of the cross, as seen by the multiple poles in the current plot in Fig. 3(D).

One of the major differences with the cross/complementary-cross structure, when compared to the cross/cross, is the presence of the lower conducting layer. The image currents in this layer, which are out of phase with the current in the upper cross, result in an “antisymmetric” nature to currents of both the dipole and quadrupole modes of the cross (see insets of Fig. 3(C,D)). In analogy to the cross/cross system, these distinct current distributions lead to the formation of current loops between upper and lower elements and their associated magnetic moments. In rotated cross/c-cross structures, we see that the presence of the c-cross in the lower sheet perturbs the image currents, yielding rotated fields, and resulting in parallel components of electric and magnetic dipoles, thus satisfying Eq. 1, the condition for optical rotation. Similar to the cross/cross system, we have competition between resonances that act to rotate the plane of polarisation in opposite directions, giving rise to our region of pure optical rotation between the resonance minima. In contrast to the cross/cross case, this region of pure optical rotation coincides with a resonant maximum in transmission. This highlights a distinct advantage of this cross/c-cross geometry over those studied in refs 4,8, 9, 10, the latter intrinsically characterised by enhanced optical reflection (and reduced transmission) associated with the closely spaced resonances of the disconnected bilayer arrays.

### Changing the Element Separation

We have seen that the optical rotation in cross/cross and cross/c-cross metamaterials has fundamentally different origins. To summarize: in the cross/cross system, electric dipoles in the upper and lower crosses couple together, forming a symmetric and an antisymmetric pair that define our transmission minima. In the case of the cross/c-cross, the transmission minima are characterised by the dipolar and quadrupolar modes of the cross. Since the transmission minima in the cross/complementary-cross structure appear intrinsic to the cross layer alone, we predict that the bandwidth of optical rotation should be relatively independent of layer separation.

**Figure 4:**
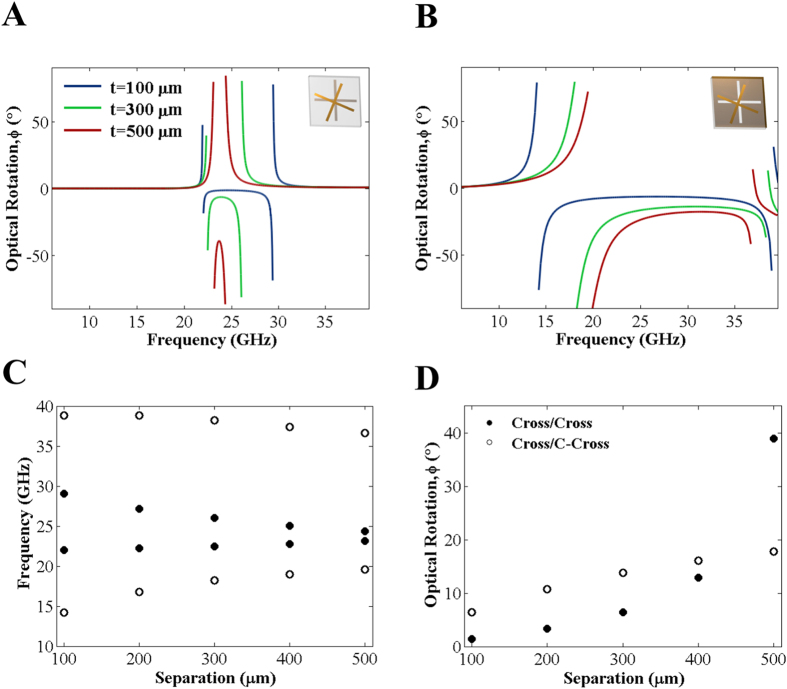
Simulated demonstration of the change in optical rotation on increasing the separation between top and bottom layers. (**A**) Modelled optical rotation for a cross/cross array (inset) with dielectric thicknesses of 100 μm to 500 μm; (**B**) Modelled optical rotation for a cross/complementary-cross array (inset) with dielectric thicknesses of 100 μm to 500 μm; (**C**) Upper and lower frequencies of asymptotes in optical rotation for cross/cross (filled circles) and cross/complementary-cross (unfilled circles) arrays; (**D**) Minimum pure optical rotation for a range of separations given by a cross/cross and cross/complementary-cross array.

In order to examine this effect further, we have carried out numerical modelling of both systems as a function of layer separation (Fig. 4(A,B)). Figure 4(C) illustrates the frequencies of the asymptotes in optical rotation for cross/cross (filled circles) and cross/c-cross (unfilled circles) structures with various thicknesses of dielectric. It is clear that the bandwidth of the pure optical rotation for the cross/cross is significantly smaller than that of the cross/c-cross for most separations. Only for very small separations do they become comparable. The magnitude of optical rotation for the cross/c-cross is also considerably larger, except for the largest layer separations (note that the large optical rotation for the cross/cross system with large layer separation is somewhat artificial, as the bandwidth is so narrow leading to a frequency overlap of the resonance modes and strong dispersion in the optical rotation). For the cross/c-cross, the magnitude of optical rotation is less dependent, in relative terms, on layer separation. Moreover, the bandwidth of the cross/c-cross is only weakly dependent on separation (interaction between the upper cross and the lower conducting sheet clearly plays a minor role in determining the resonant frequencies of the modes). Overall, this gives rise to chiroptical behaviour of the cross/c-cross which is much less sensitive to changes in the separation between upper and lower layers. These differences in the behaviour of the two systems highlight again the diverse origins of the pure optical rotation exhibited by both. In addition, this insight provides useful information for the selection of parameters for a variety of applications. If moderate optical rotation is required over a wide range of frequencies, the cross/c-cross is the most appropriate structure; however to achieve very large values of optical rotation a cross/cross structure is more suitable, although compromises in the bandwidth and transmitted intensity must be made.

## Conclusions

In summary, we have experimentally and computationally studied optical rotation in twisted cross metamaterials, comparing two distinct cases: a bilayer structure comprising of arrays of metallic crosses where the crosses in the second layer are twisted (cross/cross), and the case where the second layer is instead and array of complementary crosses (cross/c-cross). In both structures, pure optical rotation occurs in a frequency band between two transmission minima, where alignment of electric and magnetic dipole moments occurs. In the cross/cross metamaterial, the transmission minima occur at the symmetric and antisymmetric resonances of the coupled crosses. By contrast, in the frequency range of interest, we show that optical rotation region in the cross/complementary-cross structure is bounded by transmission minima associated with the dipole and quadrupole modes of the cross. Since the transmission minima in the cross/complementary-cross structure appear intrinsic to the cross layer alone, the bandwidth of optical rotation is found to be relatively independent of layer separation. This gives rise to a broad region of pure, near dispersionless optical rotation, in contrast to the cross/cross metamaterial, spanning a range from 19.0 GHz to 37.7 GHz. Our results suggest that chiral metamaterials which also include the complement to the conducting element in the unit cell (such as our cross/c-cross structures) are better candidates for optical rotatory materials in the transmission geometry.

## Methods

### Simulation

The finite element modelling in this report is performed using ANSYS HFSS software^16^. A box of a height greater than one wavelength is constructed using master/slave boundary conditions in the *x*- and *y*- directions to create an effectively infinite sample. On parallel faces Floquet ports are employed to launch and detect linearly polarised plane waves above and below the sample respectively, and allow us to determine the polarisation dependent complex transmission coefficients. The metallic elements are modelled as thin perfect electric conductors. Adaptive meshing is used to ensure each model converges appropriately.

### Sample Fabrication

The experimental cross/c-cross sample was designed using R scripts in RStudio and fabricated via traditional photolithography etching techniques by Graphic PLC. The sample consists of an Isola Tachyon printed circuit board of thickness 0.406 mm clad on both sides with 18 μm copper.

### Measurement

In the experimental setup, linearly polarized microwave radiation impinges at normal incidence upon the sample from a rectangular waveguide horn antenna that is positioned at the focus of a spherical mirror to generate a near-collimated beam with approximately planar wavefronts. The transmitted beam is collected by a second rectangular horn placed at the focus of a second mirror. Both antennas are connected to a vector network analyser (Anritsu MS4644A), allowing us to collect complex S-parameters, and can be azimuthally rotated by 90° to permit the polarisation-dependent measurements required.

### Data availability

All data created during this research are openly available from the University of Exeter’s institutional repository at: http://hdl.handle.net/10871/22113.

## Additional Information

**How to cite this article**: Barr, L. E. *et al*. On the origin of pure optical rotation in twisted-cross metamaterials. *Sci. Rep.* **6**, 30307; doi: 10.1038/srep30307 (2016).
